# Ileal Pouch-Anal Anastomosis in the Older Adult: a Review of Postoperative Outcomes and Pouchitis Treatment

**DOI:** 10.1007/s11938-022-00405-x

**Published:** 2022-12-06

**Authors:** Sabrina L. Chen, Adam S. Faye, Shannon Chang

**Affiliations:** 1 Department of Gastroenterology, New York University Grossman School of Medicine, 305 East 33rd Street, NY 10016 New York, USA

**Keywords:** Proctocolectomy, IPAA, Ulcerative colitis, Older adults

## Abstract

**Purpose of Review:**

Ileal pouch-anal anastomosis (IPAA) has become the preferred surgical treatment for patients with medically refractive ulcerative colitis (UC). Previous studies have suggested that outcomes of this procedure may be worse in older patients; however, more recent reports have suggested that IPAA in select patients is safe, feasible, and results in good quality of life. In this review, we discuss the recent literature surrounding clinical considerations and treatment management of IPAA in older adults.

**Recent Findings:**

IPAA complication rates and adverse events are similar in the older adult population, as compared to the younger adult patient population. Although fecal urgency and incontinence may be more common among older adults, chronological age alone is not a contraindication for IPAA surgery, as good quality of life can still be achieved. In this review, we will also discuss the development of pouchitis after IPAA, particularly among older adults, as the emergence of newer biologic drugs has shifted the treatment landscape.

**Summary:**

IPAA can be a safe and effective treatment modality for older adults with UC, with high self-reported patient satisfaction. Patient optimization and careful case selection are vital to achieving these outcomes, and specialized preoperative assessments and counseling can help facilitate the proper treatment.

## Introduction

Restorative proctocolectomy with ileal pouch-anal anastomosis (IPAA) has become the surgical treatment of choice for medically refractive ulcerative colitis (UC) since its introduction in 1978 by Parks and Nicholls [1]. IPAA is typically performed in a two- or three-stage procedure and allows for restored bowel continuity and avoidance of permanent ileostomy [2]. Advanced age should not by itself be used as an exclusion criterion for IPAA [3], as IPAA in older patients has been shown to be safe and effective [4–7]. However, select studies have found an increase in the frequency of postoperative complications such as infection, incontinence, and pouch failure, in older versus younger patients [8, 9].

Although there is no clear cutoff for defining “older adults” in the inflammatory bowel disease (IBD) literature, most studies have used an age cutoff ranging from 50 to 70 [10–12]. Currently, patients aged 60 years and older make up 20 to 30% of the IBD population, as well as up to one-third of all new cases [10, 13]. In addition, the prevalence of IBD among older individuals appears to be incrementally rising by 5.2% annually [14]. Still, diagnosis is often challenging in these patients, as older patients are more likely to have additional conditions that can mimic symptoms of IBD: colorectal cancer, ischemic colitis, segmental colitis associated with diverticulosis, radiation enteritis or colitis, or microscopic colitis [12]. Furthermore, the older adult population is often underrepresented in clinical trials and treatment outcome data, limiting our evidence based knowledge surrounding medical and surgical treatment options [15, 16].

It is becoming apparent that IBD treatment in the older adult population requires special consideration, particularly surrounding surgical management and medical therapy. Late-onset IBD is generally characterized by a predominance of colonic disease, with milder disease course and less frequent extraintestinal manifestations [16], although some studies have shown that a significant portion of older adults present with aggressive disease [10, 17]. In addition, these patients have an increased risk of mortality due to comorbidities, polypharmacy, and surgical complications [10, 15–18]. Furthermore, recent studies have demonstrated that rates of surgical interventions were higher in older-onset than younger-onset UC [19]. It is unclear whether the higher rate of surgical interventions in older-onset UC is driven by a less benign natural disease course, reluctance of physicians to use immunomodulators, aggressive treatment regimen in older patients, or complications due to comorbid conditions [19]. In this review, we discuss the current clinical considerations and treatment management of IPAA in older adults. Specifically, we will review postoperative outcomes and pouchitis, focusing on more recent treatment advancements for these issues.

## Special considerations for IPAA among older adults

### Postoperative outcomes

The American Gastroenterological Association and the American Society of Colon and Rectal Surgeons suggest that age alone is not an absolute contraindication to pouch surgery; rather, overall health, functional status, and comorbidities of the patient should be considered [3, 12].

A 2021 meta-analysis of 13 studies from 1996 to 2018 comparing 1124 older-onset (age cutoffs ranging from 50 to 65 years of age) with 136 younger-onset IBD patients found that increasing age did not increase rates of short- or long-term outcomes after IPAA including 30-day morbidity, 30-day mortality, pouchitis, incontinence, and pouch failure [20]. Postoperative anastomotic leak and pelvic sepsis (encompassing general sepsis, pelvic abscess, and other pelvic sepsis) are two of the most concerning complications following IPAA due to greatly increased risk of pouch failure [21]. Subgroup analysis comparing patients younger than 50, 50 to 65, and older than 65 found no significant differences among groups, suggesting that increased chronological age does not increase the risk of these two complications. These findings are consistent with other cohort studies that report age is not a significant predictor of pouch failure [22, 23].

However, a separate cohort study with 601 patients aged older than 50 who underwent IPAA for UC reported that wound infection increased with age (*p* = 0.023), though there was no increased risk of post-op fistula or pouchitis (*p* = 0.052 and 0.055, respectively) [4]. These adverse outcomes were found to be independently associated with an increased rate of pouch failure found in the 70-year-old and older patients compared with 50–59 and 60–69-year-old patient groups. A stapled anastomosis has been recommended as the standard of care whenever possible in these high-risk patients, as it has been shown to decrease the risk of infection and increase the chance of pouch retention as compared to a handsewn anastomosis [4]. Importantly, in this study and several other studies, there was no difference observed in key metrics such as daily bowel frequency, readmissions, or quality of life scores following IPAA surgery among the older adult IBD patient population as compared to younger adults (Table 1) [4, 5, 20, 24–26].

### Comorbidities

Multiple studies have found that, as expected, older patients undergoing IPAA surgery had a higher rate of systemic comorbidities (in particular, cardiovascular and respiratory diseases, but also diabetes and renal insufficiency) than younger patients [11, 25]. Older patients are also more likely to suffer from cancer (particularly colon and prostate) which may impact IPAA surgical outcomes [26, 27]. Furthermore, older patients have a higher chance of prior abdominal and pelvic surgery which can lead to longer operating times as well as higher conversion rates from laparoscopic to open procedures [25]. Some authors have attributed the presence of these comorbidities in older adults as a cause of increased short-term postoperative complications, length of hospital stay, and operating time [11, 25, 28].

However, with regard to long-term functional outcomes following IPAA, previous studies have found no differences among age groups, despite differences in comorbidities. McKenna et al. reported that patients > 50 years old had higher American Society of Anesthesiology scores (ASA) as well as increased rates of obesity and dysplasia at the time of initial colectomy as compared with patients 50 years old and younger [26]. Despite this, long-term outcome measures such as pouch failure and quality of life were similar between the two groups across the follow-up period of 5 years.

### Bowel frequency and incontinence

Functional outcomes such as bowel frequency and incontinence are important for overall quality of life following IPAA. A 2021 meta-analysis of 13 studies found that patients over 50 years old at the time of IPAA reported six to seven bowel movements daily at a pooled follow-up time of 62 months post-IPAA [20]. These results are comparable to other studies related to bowel frequency in younger patient populations [29, 30]. The same meta-analysis found that 26% of patients aged > 65 years were incontinent compared with 14% of patients between 50 and 65 years [20]. Though this is not statistically significant, it has clinical importance in advising patients and providers that increasing age may be associated with greater rates of incontinence. Similarly, Minagawa et al. reported no difference in exacerbation of daytime or nighttime soiling between 70 post-IPAA patients aged 65–69 and 66 patients aged 70 and older [5].

However, other studies, such as a 2016 meta-analysis of 12 papers comparing 4327 patients who underwent IPAA under age 50 with 513 patients 50 years or older, reported that in the first 12 months post-IPAA, individuals aged 50 and older were more likely to experience incontinence than the younger group [31]. Other cohort studies have shown that older patients have an increased risk of incontinence post-IPAA, though quality of life scores remain high in this age group [9, 24]. Lightner et al. examined a group of 1875 patients who underwent IPAA for up to 30 years after surgery and found that daytime and nighttime frequency and incontinence rates were only slightly higher in the age > 65 group compared to the overall cohort [24]. This may be related to factors independent of IPAA surgery such as pelvic floor dysfunction related to childbirth in women or muscular atrophy and surgical damage in both men and women.

Of note, surgical technique is also important when examining risk factors for increased bowel frequency and incontinence. Laparoscopic IPAA has been found to have comparable functional results to the open approach with slightly lower daytime and nighttime stool frequency [25, 32]. This difference may be related to a greater number of stapled anastomoses in laparoscopic surgery [32]. In addition, stapled IPAAs have been shown to have less daytime and nocturnal seepage, pad use, and fecal incontinence across all age groups [33].

Traditional treatment options for fecal incontinence have included conservative approaches such as lifestyle modification, dietary changes, medications such as anti-diarrheal agents, pelvic floor therapy with or without biofeedback, as well as surgery [34]. Several novel treatment options are available for post-IPAA fecal incontinence. Sacral nerve stimulation (SNS), involving subcutaneous implantation of a nerve stimulator, has been shown to benefit small cohorts of IPAA patients suffering from fecal incontinence. Seifarth et al. conducted a retrospective study of 23 patients who received SNS for increased stool frequency or fecal incontinence after proctocolectomy with IPAA for ulcerative colitis. They concluded that SNS implantation significantly improved symptoms in over two-thirds of patients suffering from high stool frequency and incontinence [35]. Similarly, Mege et al. reported improved stool and fecal incontinence/urgency following SNS in 14 out of 16 patients who previously underwent IPAA [36]. Another treatment option for patients with post-IPAA fecal incontinence is the Renew insert, an inert single-use device, which acts as an anal plug. This device was patented in 2015 and is available in various countries in Europe and Australia (currently, the device is not available in the USA). Segal et al. asked 15 patients with incontinence following IPAA to report the effectiveness of the device and found that the device was acceptable to 8/15 (53%) of patients and effective in 6/15 (40%) [37]. The device was also associated with a significant reduction in nighttime seepage (*p* = 0.034) [37].

Though in the past, older patients were not offered IPAA surgery due to concerns of fecal incontinence, recent studies have shown similar or slight increases in bowel frequency and incontinence in this population. Preoperative screening for incontinence with manometry and/or defecography in high-risk patients is recommended prior to IPAA surgery [38]. Care should be taken when deciding on surgical technique based on the surgeon’s expertise and experience, with an emphasis on stapled anastomoses. Two relatively new treatment options, SNS and the Renew Anal Plug have the potential to improve incontinence rates and quality of life in post-surgical patients. These treatment options can be considered for the older adult patient population, though most papers studying these treatments did not focus exclusively on this group. Future directions in IPAA patients may include other treatments for fecal incontinence including translumbosacral neuromodulation, which has been shown to be efficacious for patients with functional fecal incontinence without structural pathology [39].

## Pouchitis

Pouchitis, or inflammation of the ileal pouch, is the most common complication of IPAA surgery, with an estimated 70% of IPAA patients experiencing some form of pouchitis [40]. Pouchitis is clinically classified as either acute or chronic, typically with a cutoff of 4 weeks based on the duration of persistent symptoms despite therapy [41]. Chronic pouchitis can be further classified into chronic antibiotic-dependent pouchitis (CADP, favorable symptomatic or endoscopic response to conventional antibiotic therapy but with recurrent relapses that require maintenance treatment with antibiotics), chronic antibiotic refractory pouchitis (CARP, failure of symptomatic and endoscopic response to 2–4 weeks of conventional antibiotic therapy), or Crohn’s-like disease of the pouch [41]. Pouchitis requires medical therapy with antibiotics or other drugs, endoscopic and/or surgical evaluation, and intervention.

The literature surrounding pouchitis in the older IPAA population is mixed, with some studies reporting an increased rate of pouchitis in this population [4] whereas others have reported the same or decreased frequency [22, 42]. Interestingly, older age seems to be protective against the development of Crohn’s disease (or Crohn’s-like disease) of the pouch, which is a long-term inflammatory condition of the ileal pouch often requiring biologic therapy [42–44]. Though the reason for this is unclear, this may result from younger patients being exposed to a greater variety of microbial antigens as compared to older adults, leading to greater immune reactivity [45].

### Pouchitis prophylaxis

The data on using antibiotics as primary prophylaxis for pouchitis is limited. Ha published a randomized control trial of 38 patients who received either tinidazole 500 mg daily or placebo for 12 months after their final stage of IPAA surgery [46]. Results of the study showed only modest improvements in pouchitis rates, with approximately half of the patients developing pouchitis after completing the regimen. Furthermore, due to concerns with bacterial resistance and adverse effects of long-term antibiotic use, primary prophylaxis of pouchitis with antibiotics is not recommended [47]. This recommendation is especially important for the older adult population, who often suffer from polypharmacy and increased antibiotic side effects due to increased comorbidities and frailty.

Various probiotic drugs, most commonly the De Simone Formulation (formerly known as VSL#3), have been investigated for primary and secondary prophylaxis [48–53]. A systematic review that included 11 individual trials reported that the prophylaxis benefits of probiotics in pouchitis remain uncertain [54].

### Antibiotics

The first-line treatment for initial episodes of acute pouchitis is a 2-week course of antibiotics, most commonly ciprofloxacin or metronidazole [55, 56]. Typically, both medications are safe and tolerated well within the older adult patient population; however, it is important to consider the increased risk of tendon rupture with ciprofloxacin and the risk of neuropathy in long-term or high-dose metronidazole use [50, 56, 57]. Furthermore, even though ciprofloxacin is generally preferred over metronidazole given its better efficacy, tolerability, and safety profile in the general population [55], the US Food and Drug Administration warned about the risk of aortic dissection with quinolone use in older patients [58].

A meta-analysis of 21 papers on the treatment of chronic pouchitis reported that antibiotic therapy (including rifaximin, ciprofloxacin, metronidazole, and tinidazole) resulted in an overall remission rate of 74% (*p* < 0.001) in patients with CADP or CARP [59]. Similarly, combined therapies of ciprofloxacin and metronidazole or tinidazole have been shown to induce remission in chronic pouchitis patients, though side effect profiles for these drugs are more significant with long-term use [60, 61]. With CARP or Crohn’s-like disease of the pouch, treatment frequently requires non-antibiotic agents such as biologics.

### Steroid therapy

Oral steroids have been used as a second line for acute pouchitis and CARP. Topical budesonide can be an appropriate alternative for older patients with acute pouchitis due to its limited systemic absorption [62]. A randomized control trial of budesonide enema versus oral metronidazole for 4 weeks showed improvements in seven of 12 acute pouchitis patients in the budesonide group and seven of 14 patients in the metronidazole group, with fewer adverse effects in the budesonide group (25% vs 57% in metronidazole group) [63].

Although budesonide enema has been investigated for the treatment of acute pouchitis, its efficacy in CARP is not clear. A small case series of 20 patients with CARP treated with budesonide enema for 8 weeks demonstrated remission in 75% of patients, but further studies have not been done [64]. On the other hand, multiple trials have shown that oral budesonide is more effective than placebo in inducing remission in CARP patients [65, 66]. However, systemic steroid use can have significant side effects in older patients due to decreased clearance and increased toxicity including hyperglycemia, weight gain, osteoporosis, myopathy, altered mental status, and fluid retention [67].

### 5-ASA/sulfasalazine

5-ASA and sulfasalazine are other alternative treatments for acute pouchitis and CARP. A study of 22 acute pouchitis patients demonstrated that 73% of patients clinically improved and 63% underwent remission after 8 weeks of sulfasalazine treatment, with no adverse events or toxicity reported [68].

In a small case series of ten CARP patients, the use of oral, enema, or suppository mesalamine led to clinical response in 50% of patients and clinical remission in the other 50% [61]. Despite these small-scale trials, there remains a lack of strong evidence in current literature to support the effectiveness of mesalamine or sulfasalazine for the treatment of pouchitis [19]. Overall, expert consensus opinion does not support the use of mesalamine for chronic pouchitis, but there remains a role for mesalamine topical therapy for the treatment of cuffitis [41].

### Anti-TNF agents

Anti-TNF agents including infliximab and adalimumab have been used to treat chronic pouchitis. In the literature, infliximab has demonstrated efficacy in the treatment of CARP in multiple small case series [69–71], whereas data on adalimumab is scarcer and more varied in results. A small, randomized double-blind, placebo-controlled trial of 13 CARP patients found no significant difference in pouchitis disease activity index (PDAI) scores after a 12-week course of adalimumab, though this study was underpowered [72]. On the other hand, in a small case series of eight patients treated with adalimumab, clinical remission was seen in one (13%) patient and clinical response in five (62%) at week 8. At week 26, clinical remission was seen in one (13%) patient and clinical response in three (38%) [73]. One systematic review with meta-analysis found that infliximab or adalimumab significantly induced remission in patients with CARP with a 53% remission rate (95% CI 30–76%, *p* < 0.001) [59]. Another systematic review evaluated the efficacy of anti-TNF therapy in patients with CARP and found that the rates of short-term and long-term clinical remission were 50% (95%, CI 37–63%) and 52% (95%, CI 39–65%), respectively (Table 2) [74]. Of note, the overall rate of remission after anti-TNF induction therapy seemed to be higher in CD-like complications of the pouch compared to CARP (0.64 vs 0.1, *p* = 0.06).

Overall, it appears that anti-TNF agents provide clinical remission in close to half of patients with chronic pouchitis. Thus far, there have been no trials that examine outcomes of anti-TNF treatment in older patients with an IPAA. Still, it is important to consider the potential adverse effects of these medications that may disproportionately affect the elderly population. For example, multiple studies examining the safety profile of anti-TNF agents report higher rates of severe infections, cancer, and death in elderly patients compared to younger patients [75–77]. However, this may also be driven by underlying disease severity [78].

### Vedolizumab

In recent years, vedolizumab, a gut-specific monoclonal antibody against alpha 4 beta 7 integrins approved for the treatment of CD and UC in 2014, has been shown to be successful in treating refractory pouchitis. A 2019 multicenter US cohort trial of 83 patients with inflammatory disorders of the pouch including CD of the pouch treated with vedolizumab reported a clinical response in 59 (71.1%) patients and remission in 16 (19.3%) during a follow-up period of 1–3 years [79]. Another more recent systematic review of 7 studies with a total of 44 patients with CARP reported that 33 out of 44 patients (75%) reported clinical improvement after being treated with vedolizumab for 3 months (Table 2) [80].

Vedolizumab has been shown to be efficacious and safe in older patients. Khan et al. published that in a retrospective cohort of patients within the US national Veterans Affairs Healthcare System, the efficacy of vedolizumab was similar among young and older IBD patients, with the percentage of patients on vedolizumab and off steroids during the 6-to-12-month period after vedolizumab initiation at 46.8% and 40.1% for the younger and elderly age groups, respectively (*p* = 0.2374) [81]. Rates of hospitalization for IBD-related reasons within 1 year of medication start and rates of surgery for IBD-related reasons were similar between the young and older adults as well on vedolizumab [81]. The Long-term Italian Vedolizumab Effectiveness (LIVE) also found no significant difference in effectiveness between older and younger CD patients (59.4% vs 52.4%, *p* = 0.32), though the older UC group showed lower persistence on vedolizumab (51.4% vs 67.6%, *p* = 0.02) [82].

The EARNEST trial (Study to Evaluate the Efficacy and Safety of Vedolizumab in the Treatment of Chronic Pouchitis, NCT02790138) is a prospective, randomized control trial in patients ranging from 18 to 80 years of age comparing vedolizumab and ciprofloxacin to placebo and ciprofloxacin in patients with chronic or recurrent pouchitis. Exclusion criteria for this study include patients with CD, CD of the pouch, cuffitis, or irritable pouch syndrome. The primary endpoint was the percentage of participants who achieved clinically relevant remission at week 14, defined as modified pouchitis disease activity index (mPDAI) score of < 5 and reduction of PDAI score by > 1 from baseline. The primary endpoint was achieved in 31% of the vedolizumab group vs 10% for the placebo group with persistent significant differences in favor of vedolizumab at week 34. In addition, adverse events were higher in the placebo group, leading to treatment discontinuation in 10% vs 2% of the vedolizumab group [83].

The unique gut specificity of vedolizumab contributes to its favorable side effect profile in clinical trials such as EARNEST. Vedolizumab is an important treatment option for older patients who suffer from a higher number of comorbidities and are at increased risk of systemic immunosuppression [83–85]. Reported side effects of this drug include nasopharyngitis, headache, nausea, pyrexia, rash, and arthralgia, though none of these effects is noted to be more prevalent in the older adult population [86, 87].

### Ustekinumab

Few case reports and reviews exist describing ustekinumab use for chronic pouchitis (Table 2). Rocchi et al. published a systematic review of 86 total patients with IPAA and CD of the pouch or CARP. Clinical remission was reported in 10% of patients with CARP and 27% of patients with CD of the pouch after 1 year of treatment, with endoscopic response reported as 60% and 67%, respectively [96]. Minh et al. published a case report of 2 patients with CARP refractory to immunosuppressants and anti-TNF therapy, who underwent deep and sustained remission with ustekinumab [97]. A recent retrospective, multicenter cohort study reported 83% clinical response to ustekinumab in an antibiotic refractory patient population, with 73% of these patients previously treated with either an anti-TNF agent or vedolizumab after IPAA [98]. Another single-study cohort study noted a 50% clinical response in 24 total CARP patients [99]. There are no published studies examining the safety of this drug in older patients, but providers should take note that the drug is generally well tolerated in all age groups, with low to no added risk of malignancy or opportunistic infection [100, 101].

### Tofacitinib

Tofacitinib, a pan-JAK inhibitor, has been used to successfully treat moderate to severe, anti-TNF resistant UC, although the evidence for its use in pouchitis and CD of the pouch is minimal [102, 103]. One case study describes a 20-year-old woman with pouchitis following IPAA surgery, who was refractory to antibiotics, steroids, anti-TNF, and vedolizumab. In this patient, tofacitinib led to improvement in her symptoms as well as endoscopic activity in the pouch [104].

Tofacitinib has been shown to have an increased risk of cardiovascular events and nonmelanoma skin cancer [105]. In the post-marketing ORAL surveillance study of patients with rheumatoid arthritis with at least one cardiovascular risk factor, tofacitinib has been associated with increased risk of all-cause mortality, including sudden death, thromboembolic disease, and malignancies when compared to patients treated with anti-TNF [106]. Tofacitinib as well as the newer JAK inhibitors such as upadacitinib has black box warnings regarding these conditions and should be used with caution in the older adult population with underlying conditions.

## Conclusion

IPAA can be safely performed in older adults with UC with high self-reported patient satisfaction [5, 107]. IPAA complication rates and adverse effects are not dissimilar to those seen in young patients [31]. Moreover, IPAA results in similar rates of fecal urgency and incontinence among older adults as compared to younger adults, with good quality of life achieved [107].

In all, patient optimization and careful case selection are vital; chronological age alone is not a contraindication for IPAA surgery. In addition, preoperative patient selection with manometry or defecography for patients is recommended for patients at risk of anorectal disorders, particularly incontinence. Older patients should receive additional counseling with regard to the management of potential outcomes, including pouchitis, incontinence, medication side effects, and postoperative quality of life. Experienced, high-volume IPAA centers are also preferable to decrease postoperative complications in this unique patient population [108].

## Figures and Tables

**Table 1:** Summary of recent studies (2017–2022) assessing outcomes of patients based on age at the time of surgery

Study	Design	Total *n*	Age cutoff (young vs old)	Main outcome
Duraes 2022	Prospective cohort	601	60	“In elderly patients with ulcerative colitis (UC), ileal pouch-anal anastomosis (IPAA) may be offered with reasonable functional outcomes, and ileal pouch retention rates, as an alternative to the permanent stoma.” - Quality of life (QoL) score: 7.6 (age 50–59) vs 7.9 (age 60–69); *p* = 0.45 - Wound infection: 8.5% (age 50–59) vs 14.9% (age 60–69); *p* = 0.023 - Pouch failure: 2.5% (age 50–59) vs 4.4% (age 60–69); *p* = 0.015
Pederson 2021	Systematic review	1434	50	“Increasing age did not increase the rate of short- or long-term outcomes, including pouch failure.” - Pouch failure: 9.9% (age < 65) vs 6.3% (age ≥ 65); *p* = 0.372 - Incontinence: 14.2% (age < 65) vs 25.7% (age ≥ 65); *p* = 0.377 - Anastomotic leak: 4.9% (age < 65) vs 7.9% (age ≥ 65); *p* = 0.461 - Pouchitis: 21.2% (age < 65) vs 12.2% (age ≥ 65); *p* = 0.104
Minagawa 2020	Retrospective cohort	133	70	“In our analysis of elderly patients in the long-term period following surgery for UC, some noted fecal soiling, though QOL was largely maintained, and there were no serious effects on daily life.” - Stool freguency: 8 (age 65–70) vs 8 (age ≥ 70) episodes a day; *p* = 0.21 - Daytime soiling: 3.7 (age 65–70) vs 3.7 (age ≥ 70) episodes a day; *p* = 0.73 - Nighttime soiling: 4.6 (age 65–70) vs 3.5 (age ≥ 70) episodes a day; *p* = 0.16 - QoL score: 27 (age 65–70) vs 31 (age ≥ 70); *p* = 0.34
Mckenna 2018	Retrospective cohort	911	50	“Performing an IPAA on carefully selected patients older than age 50 years has minor, transient differences in pouch function compared with patients younger than age 50 years”. - Daytime incontinence: 40% (age > 50) vs 29% (age ≥ 50); *p* = 0.40 - Nighttime incontinence: 67% (age > 50) vs 66% (age ≥ 50); *p* = 0.94 - Pouchitis: 44% (age > 50) vs 53% (age ≥ 50); *p* = 0.51 - Pouch failure: 8% (age > 50) vs 14% (age ≥ 50); *p* = 0.26
Colombo 2017	Prospective cohort	77	65	“Restorative proctocolectomy can be performed in selected elderly patients, but there is a higher risk of postoperative complications and longer length of stay in this group.” - Grade IV Clavien-Dindo complication: 4.7% (age < 65) vs 16% (age ≥ 65), *p* = 0.04 - Length of stay: 8.5 (age < 65) vs 10.5 (age ≥ 65) days, *p* = 0.04 - Overall complications: 27.2% (age < 65) vs 32.4% (age ≥ 65), *p* = 0.4 - Pouchitis: 42% (age < 65) vs 20% (age ≥ 65), *p* = 0.2 - Pouch failure: 5.1% (age < 65) vs 5.1% (age ≥ 65), *p* = 0.85

**Table 2:** Efficacy of biologic medications in the treatment of chronic pouchitis

Study	Design	Type of chronic pouchitis	Total *n*	Ages of included patients	Clinical response	Endoscopic response
**Infliximab**						
Chandan et al. (2021)	Systematic review	Chronic antibiotic refractory pouchitis (CARP), Crohn’s disease (CD) of the pouch	311		65.7% (long-term clinical remission)	
Verstockt et al. (2019)	Retrospective cohort	CARP, CD of the pouch	33	Median age: 34.4	43.5% (long-term clinical remission)	
Huguet et al. (2018)	Systematic review	CARP, CD of the pouch	313		57% (long-term clinical remission)	
Kelly et al. (2016) [88]	Retrospective cohort	CARP, CD of the pouch	42	Median age: 24	62.6% (clinical response, reduction in modified pouchitis disease activity index, or mPDAI ≥ 2), 29.6% (remission, mPDAI > 5)	29.6% (mucosal healing)
Viazis et al. (2013) [89]	Retrospective cohort	CARP	7	Mean age ± SD: 37.1 (±7.2)	85.7% (clinical response), 71.4% (remission, cessation of diarrhea, urgency, incontinence, blood loss)	85.7% (mucosal healing)
Acosta et al. (2012) [73]	Retrospective cohort	CARP	33	21–67	43% (clinical response), 25% (remission, cessation of diarrhea, and urgency)	
Haveran et al. (2011) [90]	Retrospective cohort	CD of the pouch	32	18–65	53.8% (remission, cessation of diarrhea, urgency, incontinence, blood loss)	
**Adalimumab**						
Chandan et al. (2021)	Systematic review	CARP, CD of the pouch	311		31% (long-term clinical remission)	
Kjaer et al. (2019)	Randomized control trial	CARP	13	Mean age: 40.1 [21.9–48.0]	50% (reduction in mPDAI ≥ 2), 100% (any improvement in mPDAI)	67% (response)
Verstockt et al. (2019) [91]	Retrospective cohort	CARP, CD of the pouch	33	Median age: 34.4	38.5% (long-term clinical remission)	
Huguet et al. (2018)	Systematic review	CARP, CD of the pouch	313		37% (long-term clinical remission)	
Li et al. (2012) [92]	Prospective cohort	CD of the pouch	48	Mean age ± SD: 25.3 (±10.5)	54% (reduction in mPDAI ≥ 2), 33% (remission, mPDAI < 5)	39% (mucosal healing)
Shen et al. (2009) [93]	Prospective cohort	CD of the pouch	10	Median age: 36	76.5% (response), 41.2% (remission, resolution of pouch associated symptoms)	70.6% (response), 47.1% (mucosal healing)
Vedolizumab						
EARNEST (2022)	Randomized control trial	Chronic antibiotic-dependent pouchitis (CADP), CARP	102	18–80, mean age 40.8	51% (clinical response at week 34), 35.3% (clinical remission at week 34)	22.9% (endoscopic remission at week 34)
Chandan et al. (2021)	Systematic review	CARP, CD of the pouch	311		47.4% (long-term clinical remission)	
Gregory (2019)	Retrospective cohort	CADP, CARP, CD of the pouch	83	31–53	71.9% (clinical response), 19.3% (remission)	54.1% (endoscopic response), 17.6% (mucosal healing)
Singh (2019) [94]	Retrospective cohort	CARP	19	Mean age ± SD: 26.7 ± 12.8	32% (clinical response, ≥ 2 improvement in mPDAI scores)	74% (endoscopic response)
Verstockt et al. (2019)	Retrospective cohort	CARP, CD of the pouch	33		60% (long-term clinical remission)	
Bar (2017) [95]	Prospective cohort	CADP, CARP	20	Median age: 43	65% (clinical response, improvement in Oresland score)	
Ustekinumab						
Rocchi et al. (2021)	Systematic review	CARP	86	Median age: 35	63% (clinical response), 85% (CD of the pouch)	60% (endoscopic response)
Rocchi et al. (2021)	Systematic review	CD of the pouch	86	Median age: 35	85% (clinical response)	67% (endoscopic response)
Ollech et al. (2019)	Retrospective cohort	CARP	24	Median age: 25.6	50% (response)	
Weaver et al. (2019)	Retrospective cohort	CARP	56	Mean age ± SD: 44.1 ± 8.2	83% (remission)	29% (endoscopic response)
Minh et al. (2017)	Case series	CARP	2	22–71	100% (remission)	
